# Female sex: beyond risk prediction in mental health

**DOI:** 10.1016/j.lana.2026.101586

**Published:** 2026-07-29

**Authors:** Júlia Pasqualini Genro, Sofia de Lima Silva, Patrícia P. Bado, Cibele Edom Bandeira

**Affiliations:** a Graduate Program in Biosciences, Universidade Federal de Ciências da Saúde de Porto Alegre, Porto Alegre, Brazil; b Pontifícia Universidade Católica do Rio de Janeiro, Rio de Janeiro, RJ, Brazil

Damiano and colleagues provide one of the most comprehensive developmental models of suicide attempts to date, integrating genetic, perinatal, clinical, family, and childhood adversity factors over 15 years in a large Brazilian cohort. They reported that female sex remains one of the strongest predictors of suicide attempts, even after adjustment for genetic liability, perinatal factors, childhood adversity, cognitive function, psychiatric disorders, and family history of psychopathology and suicidal behaviour.^1^ This finding is consistent with epidemiological evidence indicating that suicide attempts are more frequent among girls and women than boys and men.^2^

GBD 2023 further demonstrated that this pattern extends beyond suicidal behaviour: anxiety disorders and major depressive disorder remain consistently more prevalent among women and are the leading contributors to the global burden of mental disorders.^3^ Regional evidence from Latin America mirrors the global pattern. A systematic review and meta-analysis found that women experienced a higher prevalence of depressive disorders than men, while depression rates increased in countries with greater gender and income inequalities.^4^

Adolescent girls in Brazil experience a two-fold higher burden of mental distress than boys across multiple indicators (Fig. 1). According to the 2025 Brazilian Public Security Yearbook, 88% of rape victims in 2024 were female and 77% younger than 14 years. In the same year, Brazil recorded 1492 femicides, the highest number since the crime was legally defined in 2015. These figures are especially pronounced among Black women, who account for 63.6% of all femicide victims, underscoring the intersection of gender, race, and socioeconomic inequality.^6^

**Fig. 1:**
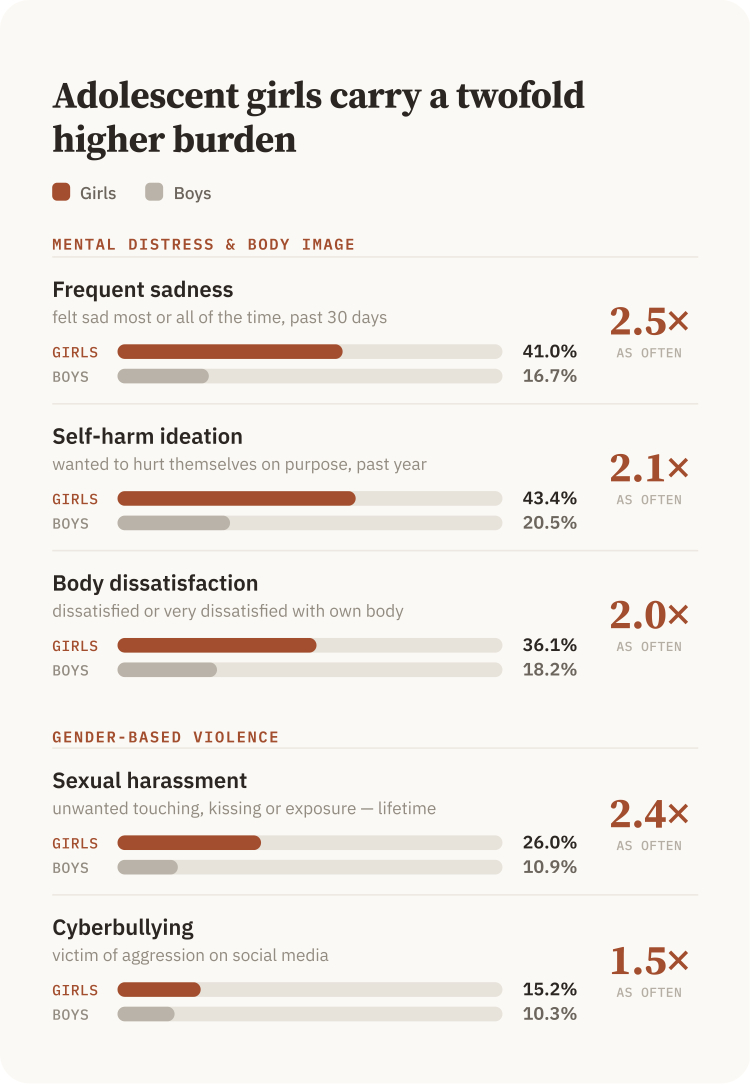
Adolescent girls experience a disproportionate burden of mental distress and gender-based violence in Brazil. Data from the 2024 Brazilian National School Health Survey (PeNSE) show that adolescent girls consistently report a substantially higher prevalence of adverse mental health outcomes and gender-based violence than boys. Data represent self-reported prevalence among Brazilian students aged 13–17 years.^5^ The ratio displayed in each panel represents the girls-to-boys prevalence ratio. Claude.ai was used to generate this figure from publicly available IBGE data; the author(s) reviewed and edited the output as needed and take full responsibility for the content.

The Brazilian findings point to a marked imbalance in the social and environmental conditions experienced by girls and women, which can be extended to the majority of countries worldwide. Across the life course, females face a disproportionate accumulation of gendered exposures, spanning everyday structural inequalities, including economic disadvantage, workplace discrimination, unpaid caregiving, and body image pressures, as well as pervasive forms of gender-based violence. These range from sexual abuse during childhood to adulthood, intimate partner violence, and, at its most extreme, femicide.^7^^,^^8^

Consequently, the observed effect of female sex may not represent a causal risk factor per se, but rather a proxy for a constellation of cumulative gender-related social exposures that remain largely overlooked in the field. Treating sex merely as a covariate or predictor may therefore obscure the role of these underlying factors in shaping mental health trajectories. This interpretation is particularly relevant for internalising outcomes that are closely linked to psychological distress, including suicidal behaviour, anxiety disorders, and depressive disorders. Notably, these psychiatric disorders, which disproportionately affect women, are also characterized by lower heritability,^9^ suggesting greater scope for environmental influences and lending further support to this hypothesis.

Despite their relevance, few studies have explicitly investigated these social determinants in an integrated manner. Longitudinal psychiatric cohorts have generated major advances in characterising genetic liability and early-life determinants of mental health, yet the systematic assessment of cumulative gender-related exposures remains comparatively limited. Reframing sex differences in this way has important implications for research and policy. It calls for the identification and quantification of specific gender-related exposures and for analytical models capable of disentangling their independent and interactive effects with biological factors across development.

This perspective aligns with the recently announced Lancet Psychiatry Commission on Women’s Mental Health, which calls for greater attention to the gendered structural determinants of mental health.^10^ If female sex captures, at least in part, the cumulative effects of these exposures across the life course, then reducing the burden of mental disorders in women will require moving beyond individual-level interventions towards sustained public policies that address these inequalities. Such policies should include educational, preventive, legislative, and protective measures, alongside broader efforts to expand economic opportunities, promote equitable caregiving, and prevent gender-based violence. It is equally important to address emerging threats, including online misogyny, technology-facilitated abuse, and harmful social media environments, which might be worsening the overall scenario.

Ultimately, a key contribution of psychiatry may lie not in confirming that women are at higher risk, but explaining why. Integrating gendered social and structural exposures with biological susceptibility across development could transform one of the field's strongest predictors into a set of modifiable mechanisms, generating the evidence needed to inform both precision psychiatry and public policies aimed at improving women's mental health across the life course.

## Contributors

JPG, PPB, and CEB conceptualized the comment and wrote the original draft, and SLS contributed to reviewing and editing the manuscript. All authors have read and approved the final version of the manuscript.

## Declaration of generative AI and AI-assisted technologies in the manuscript preparation process

During the preparation of this work, the author(s) used Claude.ai for creating the figure design. The author(s) reviewed and edited the output as needed and take full responsibility for the content of the published article. The figure was generated using Claude (Anthropic) based on summary statistics (prevalence percentages for girls and boys) drawn directly from the 5th National School Health Survey (PeNSE 2024, IBGE), a publicly available government survey. The author(s) specified the indicators, values, and the intended message of the figure; Claude generated the visual layout accordingly. The author(s) reviewed the figure for accuracy against the source data, verified all numerical values, and take full responsibility for its content and interpretation. No identifiable data were used; all figures reflect previously published, aggregate survey statistics.

## Declaration of interests

The authors declare no competing interests.
